# Spectrophotometric Analysis of Pigments: A Critical Assessment of a High-Throughput Method for Analysis of Algal Pigment Mixtures by Spectral Deconvolution

**DOI:** 10.1371/journal.pone.0137645

**Published:** 2015-09-11

**Authors:** Jan-Erik Thrane, Marcia Kyle, Maren Striebel, Sigrid Haande, Merete Grung, Thomas Rohrlack, Tom Andersen

**Affiliations:** 1 Department of Biosciences, Centre for Ecological and Evolutionary Synthesis (CEES), University of Oslo, Oslo, Norway; 2 Department of Environmental Sciences, Norwegian University of Life Sciences (NMBU), Ås, Norway; 3 Institute for Chemistry and Biology of the Marine Environment (ICBM), Carl-von-Ossietzky University Oldenburg, Oldenburg, Germany; 4 Norwegian Institute for Water Research (NIVA), Oslo, Norway; 5 Department of Biosciences, Section for Aquatic Biology and Toxicology (AQUA), University of Oslo, Oslo, Norway; CNRS, FRANCE

## Abstract

The Gauss-peak spectra (GPS) method represents individual pigment spectra as weighted sums of Gaussian functions, and uses these to model absorbance spectra of phytoplankton pigment mixtures. We here present several improvements for this type of methodology, including adaptation to plate reader technology and efficient model fitting by open source software. We use a one-step modeling of both pigment absorption and background attenuation with non-negative least squares, following a one-time instrument-specific calibration. The fitted background is shown to be higher than a solvent blank, with features reflecting contributions from both scatter and non-pigment absorption. We assessed pigment aliasing due to absorption spectra similarity by Monte Carlo simulation, and used this information to select a robust set of identifiable pigments that are also expected to be common in natural samples. To test the method’s performance, we analyzed absorbance spectra of pigment extracts from sediment cores, 75 natural lake samples, and four phytoplankton cultures, and compared the estimated pigment concentrations with concentrations obtained using high performance liquid chromatography (HPLC). The deviance between observed and fitted spectra was generally very low, indicating that measured spectra could successfully be reconstructed as weighted sums of pigment and background components. Concentrations of total chlorophylls and total carotenoids could accurately be estimated for both sediment and lake samples, but individual pigment concentrations (especially carotenoids) proved difficult to resolve due to similarity between their absorbance spectra. In general, our modified-GPS method provides an improvement of the GPS method that is a fast, inexpensive, and high-throughput alternative for screening of pigment composition in samples of phytoplankton material.

## Introduction

Quantification of phytoplankton pigments is an integral part of inland water monitoring and general experimental research involving phytoplankton. Chlorophyll *a* (chl *a*) concentrations, for example, are widely used by plankton ecologists as a proxy for phytoplankton biomass and for estimating primary productivity [1]. The relative concentrations of other photosynthetic and photo-protective pigments can provide valuable taxonomical and physiological information. Because pigment composition can be a reflection of taxonomic composition, presence or absence of certain marker pigments can be used to identify phytoplankton community composition [2, 3]. Pigment composition is also an important physiological response parameter, because the relative pigment concentrations are influenced by environmental factors such as light and nutrient availability [4].

High performance liquid chromatography (HPLC) is considered the “gold standard” for measuring pigment concentrations in plant and algal samples. HPLC can resolve most chlorophylls and carotenoids, including their degradation products such as pheophytins and pheophorbides, as long as relevant pigment standards are available [5]. However, HPLC is also expensive both in terms of time and instrumentation. Running one sample takes between 20 and 30 minutes [6], and when samples contain up to 30 different unknown pigments, the costs of standards alone can be substantial. Consequently, alternative methods for pigment quantification based on spectrophotometry are still widely used [7].

Spectrophotometric assays involve solving simultaneous equations where the unknown pigment concentrations are modeled as a function of the measured absorbance at pigment-specific peak wavelengths [8]. At best, these methods can be used to quantify chl *a*, *b* and *c* [9], total carotenoids, and pheophytins after acidification [10]. Although simple to perform, the results depend strongly on the empirical equation used. Highest precision is attained when using equations developed for pigment standards measured on the same instrument as for the unknown samples [11].

Recent spectrophotometric techniques use absorbance spectra from the whole visible region, with the aim of reconstructing the total spectrum as a weighted sum of all its individual component spectra [12]. One of these methods, termed the Gauss-peak spectra (GPS) method, represents the individual pigment spectra as linear combinations of Gaussian functions (or “Gaussian peaks”, hereafter abbreviated GPs) [13, 14]. This makes it convenient to parameterize both peak-width variations (“the widening of peaks which is observed at higher pigment concentrations due to the interaction of pigment molecules”[13]) and slight variations in pigment peak positions from one instrument to another, making the results less prone to between-instrument differences. GP spectra for 32 different chlorophylls and carotenoids were published by Küpper et al. [14], along with a spectrum fitting library for the commercial data analysis program SigmaPlot. According to the authors, the GPS method could represent a “fast, sensitive, and inexpensive alternative to analytical pigment HPLC”.

In practice, however, the method has potential for improvement. In this paper, our goal is to make the GPS method more generally available, applicable to a wider range of ecologically relevant pigments, and suitable for detection of pigments in natural samples.

## Materials and Methods

### Gaussian peak representation of individual pigment spectra

Küpper et al. [14] represent the individual pigment absorption spectra as linear combinations of GPs, an idea we have maintained in our modified version of the method. Absorption spectra can be efficiently represented by weighted sums of GP functions:

$$x(\lambda )=\sum_{j=1}^{k}b_{j}G(\lambda ,m_{j},w_{j})$$

where G is a bell-shaped function of wavelength with a single peak located at *λ* = *m* (nm) and with a half-peak width equal to *w* (nm):

$$G(\lambda ,m,w)=\exp(-\frac{1}{2}\frac{{\left(\lambda -m\right)}^{2}}{w^{2}})$$

G is functionally equivalent to the Gaussian (or normal distribution) function, just without the normalization coefficient. Further statistical details can be found in the original paper, and in Naqvi et al. [12].

### Instrument calibration

Küpper et al. [14] include two additional parameters, *δ* and *ω*, to adjust for slight variations between spectrophotometers in spectral peak positions of pigment standards (*δ*; nm) and the “widening of peaks”-effect (*ω*; a dimensionless constant):

$$G(\lambda ,m,w,\delta ,\omega )=\exp(-\frac{1}{2}\frac{{\left(\lambda -m-\delta \right)}^{2}}{{\omega w}^{2}})$$

The original method estimates a specific value for these two parameters along with the pigment and background weights for every sample, making the estimation procedure non-linear. We consider these parameters to be related to the wavelength calibration and the optics of a specific instrument, and use the spectrum from a chl *a* standard (or similar) to estimate two instrument-specific, global values for *δ* and *ω*. These two (constant) values are then to be used for all samples measured on the same instrument. This step is important, because it is a prerequisite for the downstream use of linear optimization. An R-script [15] for estimating instrument specific parameter values (calibrating the instrument) can be found in S1 File.

### Modeling of pigment and background spectra by non-negative least squares (NNLS)

NNLS is a type of linear least squares fitting, which involves finding model parameter values that minimize the sum of squared differences between observations and model predictions. If the model is a linear weighted sum of the unknown parameters, the global minimum is found in a single step by solving an over-determined system of linear equations. On the other hand, if the model is non-linear in some of the unknown parameters, the fitting is done by an iterative search where the solution may depend on the starting values. Parameters fitted by linear or non-linear least squares can be positive or negative. This property is not desirable for fitting spectra since it is physically impossible for the spectral coefficients (that is, the weight given to each pigment in the mixture) to have negative values. NNLS is a modification of the least squares algorithm where the fitted parameters are constrained to be non-negative (≥0). Lawson & Hanson [16] showed that the NNLS problem could be solved efficiently with approximately the same computational effort as ordinary least squares. Using NNLS to fit unknown pigment mixtures thus is expected to be far more computationally efficient and numerically stable than the constrained non-linear least squares method proposed by Küpper et al. [14].

**Fig 1:**
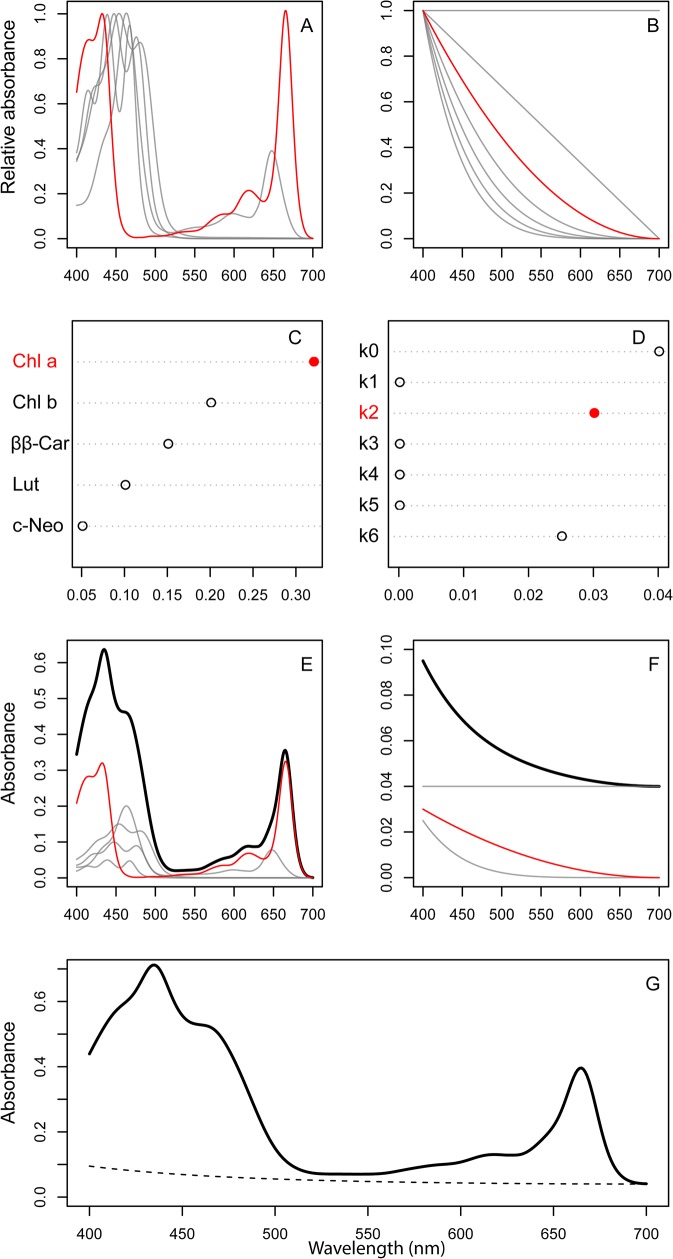
Conceptual overview of the fitting procedure. A) Individual pigment spectra (here, chl *a*, chl *b*, ββ-Car, Lut, *c*-Neo), and B) background basis spectra are assigned weights (C, D) in the NNLS regression, where the weights depend on the composition of the pigment extract. The weighted component spectra are summed to obtain the total pigment spectrum (E) and the total background spectrum (F). Adding together the pigment and background spectra yields the total spectrum of the extract (G).

The pigment spectra generated from a set of GPs are used as component spectra (Fig 1A) in the actual NNLS modeling of the measured absorbance spectra. Before this step however, we will introduce similar component spectra for modeling the background spectrum (Fig 1B). By background spectrum, we mean any light attenuation *not* attributable to pigment absorption; mainly scattering by particles in the sample, or absorption by non-algal components extracted from the sample. Küpper et al. [14] modeled the background spectrum with a 3-parameter decreasing exponential function. As a consequence this turns the spectrum-fitting problem into a non-linear one, even when using NNLS to fit the mixture component spectra. The absorbance spectrum of the non-algal background is generally found to be smooth and featureless, with an exponential decrease with increasing wavelength.

We observe that as with any continuous function, the decreasing exponential can be approximated by a Taylor polynomial. In other words, we can represent the decreasing exponential background spectrum as a linear combination of power functions of wavelength. If we construct background basis functions as powers of a linear function decreasing from 1 to 0 over the wavelength range of the fit, the coefficients of a Taylor expansion of the negative exponential will have non-negative coefficients. Consequently, we can use NNLS to fit both the background spectrum and the pigment mixture coefficients. In practice this is done by running NNLS with an augmented design matrix *X* = [*X*_*p*_ *X*_*b*_] consisting of the pigment spectrum model matrix (*X*_*p*_) and a background model matrix (*X*_*b*_) whose columns are power basis functions. We let the first column of *X*_*b*_ consist of ones, such that the fitted coefficient for this column will represent a possible constant baseline offset in the spectrum. The coefficients fitted by NNLS will thus consist of two groups, of which the former represent the pigment mixture weights while the latter can be used to construct the fitted background spectrum.

We then used the following calculations to fit the data using NNLS (for simplicity, we left out the background components from the description). If we have a mixture of *n* pigments with known absorbance spectra *x*_*i*_(*λ*), *i* = 1 … *n* at wavelengths *λ* (nm), then the spectrum of the mixture *y(λ)* will be a weighted sum of the individual component spectra:

$$y(\lambda )=a_{1}x_{1}(\lambda )+a_{2}x_{2}(\lambda )+\ldots +a_{n}x_{n}(\lambda )$$

If the component spectra are normalized to unit maximum peak absorbance, the weights (*a*_*i*_) will be equal to the product of pigment concentration (*c*_*i*_; g L^-1^) and the pigment’s weight-specific absorption coefficient at the maximum peak wavelength (*u*_*i*_; L g^-1^cm^-1^): *a*_*i*_ = *c*_*i*_ *u*_*i*_. In matrix notation, this mixture model can be written as:

y(λ)=X(λ)a

Where *X*(*λ*) = [*x*_1_(*λ*),*x*_2_(*λ*),…*x*_*n*_(*λ*)] is a matrix with columns that are the component pigment spectra, and *a* = [*a*_1_,*a*_2_,…,*a*_*n*_]^*T*^ is the column vector of mixture weights. Estimating the pigment composition of an unknown mixture by ordinary least squares then reduces to finding a mixture weight vector *â* such that the L_2_-norm of the residuals ${\left\|y(\lambda )-X(\lambda )\hat{a}\right\|}_{2}$ is minimized. Remembering that the mixture weights cannot be negative, we minimize the residual L_2_ norm subject to the non-negativity constraint *â*>0 by the NNLS algorithm.

### Calculation of individual pigment concentrations

Concentration of pigment *i* in the extract (mg/L) was further calculated as:

$$c_{i}=1000(\frac{a_{i}}{u_{i}})$$

Specific absorption coefficients (L g^-1^cm^-1^) were obtained from Appendix F in *Phytoplankton pigments–characterization*, *chemotaxonomy and applications in oceanography* [17]. If a coefficient determined for ethanol was available, it was used. If not, we used the value recommended by [17] even if it was from another solvent. Coefficients and original references ([10], [18–34]) are listed in Table 1.

**Table 1:** Pigment list.

code	Pigment	Abbrev	Abs. Coef	Abs. peak	HPLC	Source	Solute
c,p,f *	Chlorophyll *a*	Chl *a*	83.9	432	L,S,C	[10]	Ethanol
c *	Chlorophyll *b*	Chl *b*	107	464	L,S,C	[10]	Ethanol
*	Chlorophyll c_1_	Chl *c*_1_	318	443	L,S	[18]	Acetone
f *	Chlorophyll c_2_	Chl c_2_	374	444	L,S,C	[18]	Acetone
c p f *	Pheophytin *a*	Phe *a*	143	417	L,S,C	[10]	Ethanol
c *	Pheophytin *b*	Phe *b*	141	437	L,S,C	[10]	Ethanol
p *	β,β-Carotene	ββ-Car	262	453	L,S,C	[19]	Ethanol
f *	Alloxanthin	Allo	216	464	L,S,C	[20]	Benzene
p *	*trans*-Canthaxanthin	Cantha	220	469	L,S,C	[21]	Cyclohexane
*	*trans*-Diadinoxanthin	Diadino	224	448	L,S,C	[22]	Acetone
*	Diatoxanthin	Diato	272	453	L,S,C	[23]	Acetone
*	Dinoxanthin	Dino	210	442	L,C	[22]	Acetone
p *	*trans*-Echinenone	Echin	216	458	L,C	[24]	Hexane
*	Fucoxanthin	Fuco	166	443	L,S,C	[23]	Acetone
c *	Lutein	Lut	255	447	L,S,C	[25]	Ethanol
p *	Myxoxanthophyll	Myxo	216	478	L,C	[26]	Acetone
*	9’-*cis*-Neoxanthin	*c*-Neo	233	437	L,C	[27]	Ethanol
*	Peridinin	Peri	135	475	L,C	[28]	Ethanol
c *	Violaxanthin	Viola	254	437	L,C	[29]	Ethanol
/	Antheraxanthin	Anth	235	446	L,C	[30]	Ethanol
/	*cis*-Canthaxanthin	*c*-Cantha	220	269	-	[31]	Cyclohexane
/	β -Cryptoxanthin	Cryp	247	453	-	[32]	Ethanol
/	*cis*-Diadinoxanthin	*c-*Diadino	224	448	-	[23]	Acetone
/	*cis*-Echinenone	*c*-Echin	211	461	-	[21]	Cyclohexane
p f /	alll-*trans* Zeaxanthin	Zea	245	453	L,S,C	[32]	Ethanol
/	9-*cis*-Zeaxanthin	9-*c*-Zea	245	450	-	[33]	Diethyl ether: methylbutane:ethanol 5:5:2
/	13-*cis*-Zeaxanthin	13-*c*-Zea	245	450	-	[33]	Diethyl ether: methylbutane:ethanol 5:5:2 5:5:2
/	Pheophorbide *a*	Pheide *a*	177	411	S	[34]	Tetrahydrofuran

Pigments with abbreviations (Abbrev) used for the modified Gaussian Peak Spectra (GPS) method described in this paper. Specific absorption coefficients (Abs. Coef; L g^-1^ cm^-1^) and corresponding absorbance peaks (Abs. Peak; nm) are given. The HPLC column indicates if a standard for the pigment was included in the HPLC analysis of the sample

L: lake samples

S: sediment

C: cultures.

Pigments (code) are identified as follows:

* = “core” set

c = culture specific set (*Chlamydomonas*

p = Planktothrix

f *= Cryptomonas*

Pigments not included in the final “core” set (/) due to high error rates.

### Development of a core set of pigments for unknown samples

Küpper et al. [14] presented GP parameters describing the absorption spectra of 12 chlorophylls and 20 carotenoids. We updated this list with four new pigments from common phytoplankton species: peridinin (peri), dinoxanthin (dino), alloxanthin (allo), and pheophorbide *a* (pheide *a*; refer to Table 1 for pigment names and abbreviations). After obtaining the absorbance spectra for the new pigments (described below), they were fitted as sums of GPs (see S2 File for an R-script describing the estimation). We also obtained new spectra and GPs parameters for chl *a* and *b* in ethanol. We removed eight uncommon pigments from the original list of Küpper et al. [14], including all zinc, copper and cadmium chlorophylls, diadinochrome, and aurochrome, to obtain a list of GP parameters for 28 of the normally occurring pigments in natural phytoplankton communities.

A general problem with spectral deconvolution techniques, also mentioned by Küpper et al. [14], is aliasing. In other words, since some pigments have extremely similar absorption spectra, there might exist several weighted combinations of component spectra that can add up to approximately the same total spectrum. When measuring samples of unknown community composition (for example lake samples), many pigment component spectra need to be included to capture what might be present. However, if spectra are easily confused, it might be better to only include a “core set” of pigments that are less prone to aliasing for such samples. We made decisions on which pigments to include in such a “core” based on a Monte-Carlo simulation of each pigment’s identification error rate.

The simulation was done as follows: A simulated pigment mixture was developed by randomly selecting four pigments and combining their GP spectra to create a test sample. This spectrum was scaled to a peak absorbance of 1 and perturbed with normally distributed white noise with a certain standard deviation (Sd). The simulated mixture-spectrum was then fitted by NNLS using a pigment spectrum model matrix (*X*_*p*_) containing all the 28 different pigment spectra. These steps were repeated 100,000 times and used to calculate frequencies of detecting a pigment that was not present in the test sample (false positive), and of failing to detect a present pigment (false negative). This procedure was again repeated for 10 different values of Sd (0.00001, 0.00003, 0.00007, 0.0002, 0.00055, 0.0015, 0.0041, 0.011, 0.03, 0.082, and 0.22). Increasing the standard deviation of the errors in a stepwise fashion made it possible to assess both the algorithm’s sensitivity to experimental error, and the differences between pigments in error rates. The R scripts used for these simulations are included in S3 File.

### Sample preparation and analysis of absorbance spectra

#### Lake, sediment, and culture samples

Samples of natural phytoplankton communities were taken from 75 lakes (described in [35]) and pigments were extracted in 96% ethanol overnight at 4°C in the dark. All lakes were located on public land, and prior to sampling permission was obtained from local Norwegian municipalities: Aremark, Arendal, Aurskog-Høland, Trøgstad, Bergen, Bjerkreim, Bjugn, Bærum, Oslo, Elverum, Fet, Rælingen, Enebakk, Trøgstad, Flesberg, Fræna, Fusa, Gjesdal, Grue, Halden, Hemne, Hjelmeland, Hof, Hurum, Jevnaker, Gran, Søndre Land, Dokka, Jølster, Krødsherad, Flå, Larvik, Levanger, Lunner, Jevnaker, Løten, Marker, Nord-Aurdal, Nord-Aurdal, Vestre Slidre, Oppegård, Ringerike, Ringsaker, Rømskog, Sandefjord, Larvik, Sandnes, Skaun, Skodje, Haram, Stange, Stavanger, Randaberg, Strand, Stryn, Sveio, Søndre Land, Sør-Odal, Nord-Odal, Tokke, Tokke, Kviteseid, Trysil, Tysvær, Vestre Toten, Vindafjord, Vinje, Volda, Voss, Ørsta, Åmot, Trysil, Åsnes. No endangered species are present in any field site.

Samples for sediment pigment analyses were obtained from a core taken at 25 m depth in Lake Steinsfjorden (60°05'43.17"N 10°19'30.84"E). The core was sliced in 1 cm sections starting from the sediment surface and maintained at -20°C. Prior to extraction, the samples were freeze-dried; subsamples were placed into pre-weighed 5mL polypropylene vials and re-weighed. Ethanol (96%) was added by pipette and the entire tube–sample and extraction solution–was again weighed to obtain the amount of ethanol used for extraction. Extraction volumes were then calculated from weights, assuming a mass density of 96% ethanol of 0.81 g mL^-1^. Sediment to extraction volume was approximately 0.4–0.6 g mL^-1^. Samples were thoroughly mixed by vortex and followed by gentle centrifugation to ensure all sediment remained in contact with the solvent. The samples were then extracted for 20 hours in the dark at 4°C.

Cultures of *Planktothrix* (including both red and green strains), a filamentous cyanobacterium, were obtained from the Norwegian Institute of Water Research Culture Collection and harvested during the exponential growth phase from batch cultures grown at 20°C under 3–5 μmol PAR m^-2^s^-1^, in Z8 medium. Aliquots of the cultures were centrifuged, and pellets transferred to vials and lyophilized. The freeze-dried samples were weighed, and then extracted in 96% ethanol overnight at 4°C. Cultures of the chlorophyte *Chlamydomonas reinhardii* (strain cc-1690) and the cryptophyte *Cryptomonas ozolinii* were harvested at the end of the exponential phase from batch cultures grown at a light level of approximately 35 μmol PAR m^-2^ s^-1^. Ten milliliters of each culture was filtered onto 25 mm Whatman GF/F filters, and stored at -80°C until extraction in 3 mL 96% ethanol overnight at 4°C.

#### High-throughput absorbance measurements

All absorbance (that is, ${log}_{10}(\frac{I_{0}}{I})$) measurements were made in a Synergy MX plate reader (BioTek instruments, Vermont, USA) and all spectral scans were recorded between 400 and 700 nm using one nm resolution. Because the path length for a top-reading plate reader is dependent on the volume of liquid in the chamber, the path length (l, cm) in the micro-well was calculated as v/a, where *v* (cm^3^) is the volume of the sample and *a* (cm^2^) is the area of the well bottom. Absorbance was, however, *not* normalized to unit cm^-1^ until after pigment weights were estimated by NNLS. The absorbance spectra were saved as a data matrix with one sample spectrum per column, and used as input to the R-script in the downstream analysis. Note that the absorbance of a blank (plate and solvent absorbance) was not subtracted. Instead, this absorbance was modeled as a part of the general background, as described above.

For the spectral scans, different well plates and well volumes were used depending on sample-type. Sediment extracts utilized 96-well flat bottom clear polypropylene plates (Greiner bio one #655201) with 330μL sample per well. For cultures, 96-well polystyrene plates (Greiner bio one, μClear F-bottom) with 330 μL of sample volume were used. For natural lake samples, we used 48-well polystyrene plates (Nunc flat bottom, Thermo scientific) with 750 μL per sample.

To assess possible background effects of different micro-plate material, we utilized one extract from a single culture (*Planktothrix*) and scanned using three different types of 96-well-plates: clear polystyrene (Greiner bio one), white polystyrene with clear bottom (Greiner bio one, μClear, F-bottom), and clear polypropylene (Greiner bio one, F-bottom). Pigment and background spectra were fitted with NNLS and final pigment concentrations compared (S2 Fig).

#### Use of ethanol as solvent

Küpper et al. [14] used acetone for pigment extraction. Because acetone is highly volatile, samples evaporated rapidly from our wells and made a full 96 well plate difficult to measure in one run. Another possible extraction solution was methanol, also commonly used in pigment analysis. However, both acetone and methanol have negative effect on pipettes [36] and they require the use of polypropylene micro-plates. For these reasons, the solvent was changed to 96% ethanol. Ethanol has been shown to be an effective extraction solvent in pigment analysis [36]. While each solvent has its own virtue, we selected ethanol to allow for ease of 96 well plate usage and for occupational safety. No further comparison of extraction efficiency was done for this study. However, replicate samples of one culture (*Chlamydomonas*) were extracted using either ethanol or acetone and then analyzed using either the software from Küpper et al. [14] or our modified method to compare concentration differences between the original GPS and modified-GPS.

#### HPLC analyses

Replicate samples from the natural lake and culture samples were analyzed by HPLC at Wassercluster Lunz (Austria). Pigments were extracted in 90% acetone. To improve extraction, samples were combined with quartz sand, vortexed, and sonicated for 30 minutes on ice. Samples were stored at 4°C in the dark for 24h before analysis. Analytical protocol and HPLC system was the same as in Schagerl & Künzl [37]. Sediment pigments were analyzed by HPLC at the Norwegian Institute for Water Research (NIVA). Pigments were extracted from freeze-dried samples in 90% acetone in for 4h. One minute of sonication was applied to improve extraction. Samples were centrifuged before the supernatant was injected into a HPLC system. Analytical protocol and HPLC system was the same as in Hobaek et al. [38]. Pigment standards used for detection are listed in Table 1, for both labs.

#### Measurement of additional pigment spectra

Pure standards, including chl *a* (Sigma-Aldrich), allo (DHI; Hørsholm, Denmark), and pheide a (DHI), were dissolved in 96% ethanol. Absorbance spectra (400–700 nm, every nm) were recorded using the plate reader and clear polypropylene microwell plates (Greiner bio one, F-bottom) containing 300 μL sample per well. The absorbance of a well with 96% ethanol was subtracted. Spectra for chl *b* (95% ethanol), dino (ethanol, unknown %), and peri (acetone, unknown %) were digitized from Lichtenthaler [10], Jeffrey et al. [39], and Roy et al. [17], respectively.

### Statistical analysis

#### R-scripts and data

R-scripts were developed for our modified-GPS method and can be found in S4 File, S5 File. These folders include the data and scripts used to obtain the results from the sediment and natural lake samples, respectively. Each folder includes 1) the main script with two accompanying sample text files consisting of sample information and sample scans and 2) the “pigment.function.R” script with two accompanying text files. For example, the S5 File (Lake) folder includes a main script for analysis (“Lake.R”) and two text files containing absorbance spectra from 75 lakes (“Lake abs spec.txt”) and sample IDs with filtration volumes (“Lake sample ID.txt”). The main script is used to decompose the measured spectra into pigment and background components, and to calculate pigment concentrations. The script “pigment.function.R” must be present in the folder so that it can be loaded with the main script (using the R function “source”) to make the fitting functions available to the user. In short, these functions, which are further described in S1 Text, include the “pigment.basis” function that generates the pigment basis spectra from their GP parameters (Fig 1A). The “background.basis” function creates the background basis spectra as powers of a linear function decreasing from 1 to 0 over the wavelength range of 400–700nm (Fig 1B). The “pigment.fit” function does the actual NNLS fitting of the measured absorbance spectra as weighted sums of basis spectra (Fig 1C & 1D). The three functions “pigment.spectrum”, “background.spectrum”, and “fitted.spectrum” use the results from “pigment.fit” as input, and calculates the total pigment spectrum (Fig 1E, black line), the total background spectrum (Fig 1F, black line; g, dashed line), and the total fitted spectrum (Fig 1G, solid line), respectively. The last function “pigment.concentration” also uses “pigment.fit” as input, as well as a path-length (L, cm) for absorbance normalization. It yields concentrations of all the detected pigments as mg L^-1^ in the extract. Note that any conversion to sample concentrations (for example μg L^-1^ of lake water), must be done afterwards. The “pigment.function.R” script needs two text files that include the GP parameters (“gaussian.peak.parameters.txt”) and the weight-specific absorption coefficients (“specific absorption coefficients.txt”). Both of these must be contained in the same folders as the other R files.

#### Comparing pigment concentrations with HPLC results

Pigment concentrations obtained by the modified-GPS method were compared with results from HPLC. In the first level of pigment comparison, we pooled all chlorophylls and all carotenoids from both the modified-GPS and HPLC results. The resulting total chlorophyll (total chl) and total carotenoid (total car) concentrations were compared using linear regression within the natural lake samples (n = 75) and sediment samples (n = 40).

Rather than comparing each pigment by correlation or linear regression, we used principal component analysis (PCA) to compare the results from the two methods on single-pigment level. We did so because not all pigments were present in both methods (not all HPLC standards were available as GP spectra, and vice versa), and because of the tendency of the GPS methods to confuse carotenoid spectra. If the two methods identified similar gradients in pigment composition, the PCA axes calculated from the two pigments vs. sample matrixes should be correlated. We used Kendall’s τ as a measure of correlation due to non-normal axis scores. For the PCA on natural lake samples, some pigments were combined. These included chl *a* with phe *a*, chl *b* with phe *b*, and chl *c*_*1*_ with *c*_*2*_ which then created three different totals (tot chl *a*, tot chl *b*, and tot chl *c*). All pigment concentrations were normalized to the total chl *a* concentration. In the PCA on sediment samples, all pigment concentrations were normalized to tot chl *a*, calculated as the sum of chl *a* and phe *a*. Some pigments were not present as both GP spectra and HPLC standards, such as α-carotene, 19’-butanoyloxyfucoxanthin, crocoxanthin, and lycopene, which were present as HPLC standards, but not as GP spectra. These pigments were omitted from the PCA. For culture samples, individual pigment concentrations were compared directly. To assess the method’s ability to reconstruct measured absorbance spectra, we calculated root mean squared errors (RMSEs) between observed and fitted spectra.

#### The extent of background attenuation

We assessed the contribution of the background spectrum to the total spectrum in natural lake and sediment samples by fitting absorbance spectra with the solvent blank subtracted as weighted sums of the component spectra. Any background signal estimated for these spectra should be due to either scattering, or absorption by non-algal components extracted from the filter. In addition, we related the extent of these background spectra (the integral under the spectrum from 400–700 nm) to the amount of particulate organic carbon (POC) and chl *a* in the natural lake samples (described in [35]). We hypothesized that a high POC:chl *a* ratio could lead to an increased background contribution.

#### Direct comparison of GPS and modified-GPS

A test to compare original GPS and modified-GPS methods was done using extracted pigments from the same sample with two different extraction methods, either acetone and ethanol, matched with the appropriate standards for each solvent. A green algal culture (*Chlamydomonas*) was used with duplicate replicates for each method. We applied Küpper et al.’s [14] method (the “green algae” module in their SigmaPlot program) on the acetone extracts and our version (with chlorophyte pigments as candidate pigments) on the ethanol extracts.

## Results

### Aliasing and selection of a core pigment set

**Fig 2:**
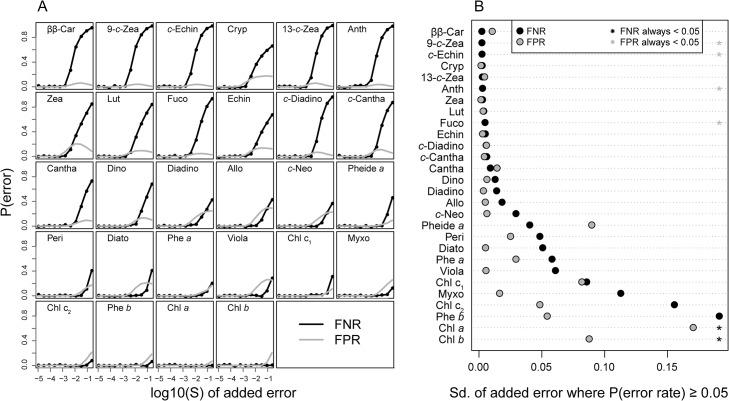
Results from the Monte-Carlo simulation of pigment detectability. In A), the course of the false negative rate (FNR; black line) and the false positive rate (FPR; grey line) as the standard deviation of the random error is increased (see text), is shown for each pigment. The pigments were sorted from highest to lowest FNR, also shown in B). Here, we estimated how high the added random noise had to be for the FNR and FPR to exceed 0.05 (5%). Black dots represent the standard deviation of this random error for the FNR, the grey dots for the FNR. Asterisks mean that the FNR (black asterisks) or FPR (grey asterisks) never exceeded 5% for that pigment.

The results from the Monte-Carlo simulation showed that false positive and false negative rates increased with simulated measurement noise (Sd) (Fig 2A). The false negative rates were generally higher than the false positive rates (Fig 2B). At low Sd values (< ~ 0.001) most pigments were correctly identified, but some carotenoids (zea and cryp) were falsely identified even at Sd < 0.001. At Sd values between 0.001 and 0.01, most carotenoids had false positive rates close to 0.1 and false negative rates between 0.1 and 0.7 (Fig 2A), indicating a strong tendency for aliasing for these pigments. All chlorophylls had low error rates except at the highest Sd values (well above 0.01). Such noisy spectra are, however, not realistic unless the pigment concentration is very low.

The results from the simulation was used to identify a selection of “core pigments” that would be the most likely pigments to find in a natural sample, as well as having a high probability of identification. For instance, zea and cryp, were removed due to their very high false positive rates and high correlation with ββ-Car (r = 0.999). 19 pigments were selected for the final core (see Table 1) and further tested by an additional Monte-Carlo simulation indicating that these pigments were identifiable (S1 Fig). Table 1 summarizes the original 28 pigments tested and lists those that were removed to obtain the core set. GP parameters for the core pigments are used as default in the functions contained in “pigment.function.R”. Note, however, that when a specific pigment profile is expected (such as for samples from known algal cultures), it is possible to adjust the list of candidate pigments. This can be easily accomplished in the R functions (see S1 Text for an example). As noted in Küpper et al. [14], removal of unexpected pigments in a particular research question would further improve the quality of the estimates.

### Test of performance by the modified-GPS method

For the modeling of sediment and natural lake samples we use the core set of candidate pigments, while culture samples were modeled using an appropriate, species-specific subset (see Table 1).

#### Sediments

**Fig 3:**
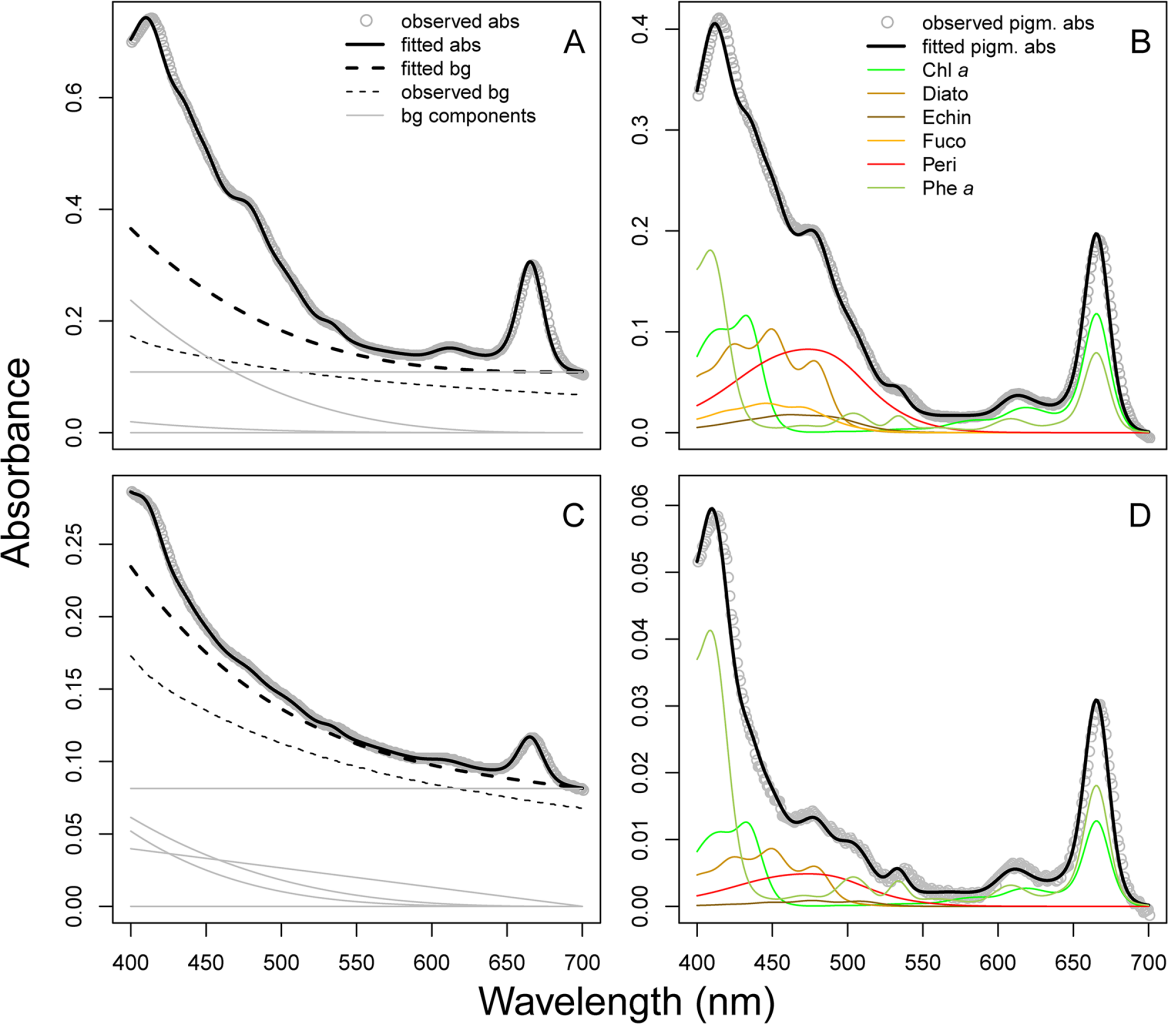
Absorbance spectra measured on sediment extracts. Data from two depths in the sediment column is shown: A) and B) are from the upper cm, C) and D) from 5 cm below the sediment surface. The difference in absorbance between the two samples can be attributed to lower pigment concentrations deeper in the sediment. A & C) The measured total absorbance spectrum (grey dots) overlaid with the fitted total absorbance spectrum obtained using the modified-GPS method (solid black line). Background spectra (scattering and/or absorbance by non-algal pigment) are represented by dotted lines; the thick line being the modelled background, while the thin line is the measured blank spectrum (absorbance of a well filled with 96% ethanol). Thin grey lines represent the different component spectra making up the modelled background spectrum. In B) and D), the modelled background spectrum is subtracted from the total spectrum, yielding only pigment absorbance. The spectra of the individual pigments recognized by the modified-GPS method are shown in colour.

**Fig 4:**
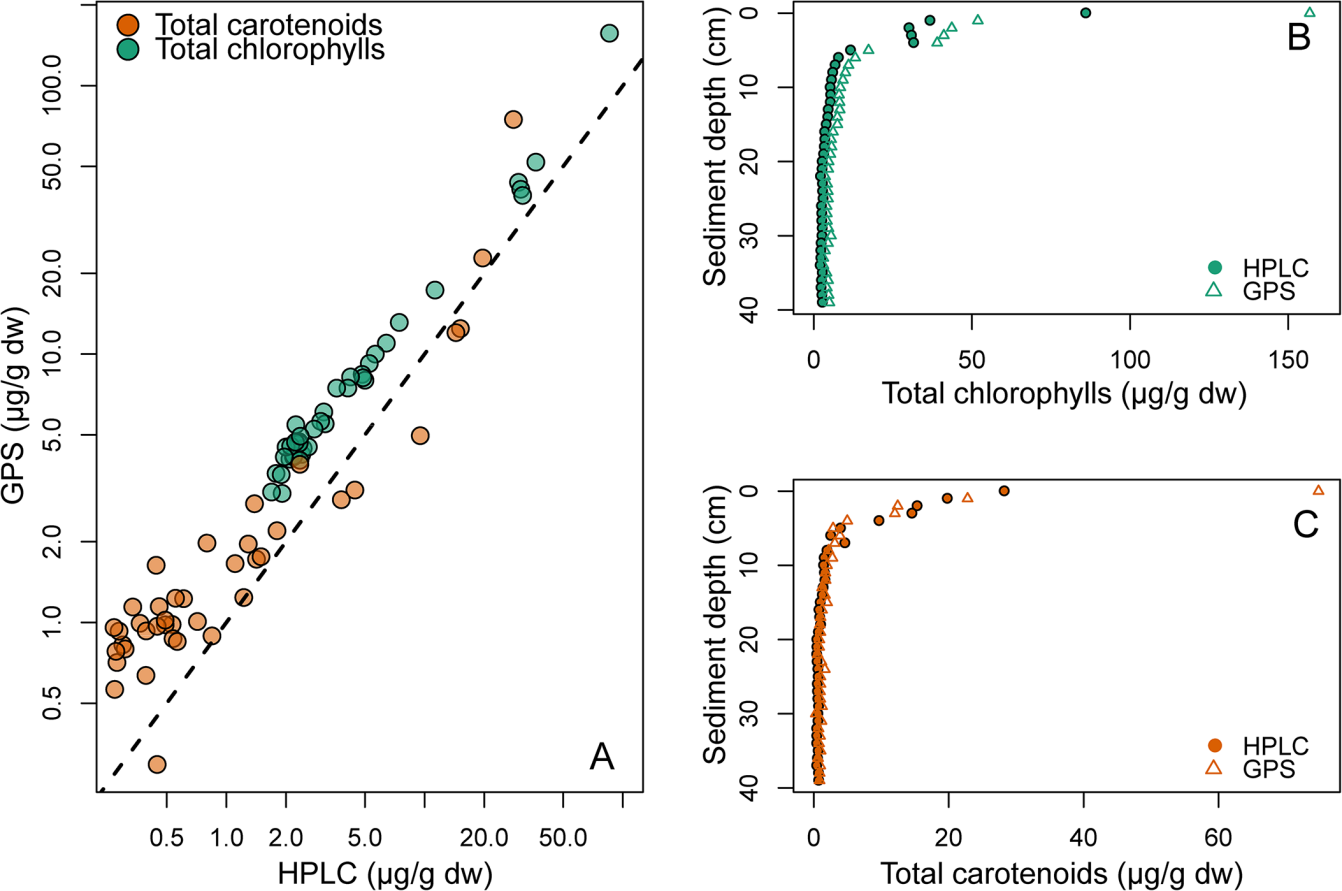
Total chlorophylls and total carotenoids from sediment. A) Concentrations (μg pigment per gram sediment dry weight) of total chlorophylls (green) and total carotenoids (orange) as estimated by modified-GPS (y-axis) and HPLC (x-axis). The dotted line shows the 1:1 relationship. Note that both axes are log-transformed due to the large variation in concentrations. Pigment concentrations decreased with sediment depth, both total chlorophylls (B) and total carotenoids (C).

The sediment absorbance spectra fitted by NNLS were very similar to the observed absorbance spectra (Fig 3). Samples deeper than approximately 5 cm had very low pigment-to-background ratio (Fig 3C), but distinct pigment spectra were still apparent after subtracting the modeled background spectrum (Fig 3D). Root mean squared error (RMSE) values spanned from 0.0004 to 0.005 with a mean of 0.00095. Concentrations of total chl (calculated as the sum of all chlorophylls and pheophytins) and total car (sum of all xanthophylls and carotenes) estimated by modified-GPS were significantly correlated with HPLC (Fig 4A, log-log relationships: r^2^ = 0.99 for total chl, and 0.86 for total car, both p-values < 0.0001, n = 40), and both methods captured the expected decline in pigment concentrations with sediment depth (Fig 4B and 4C). Absolute concentrations were generally slightly higher in the modified-GPS than HPLC for both pigment types (Fig 4). This, however, might be explained by differences in extraction procedure between the two methods. The largest difference in total car between methods was observed at low carotenoid concentrations (Fig 4A). The two first PCA axes (figure not shown) estimated from the modified-GPS results were significantly correlated with the corresponding axes from HPLC (PCA1: Kendall’s τ = 0.47, p < 0.001; PCA2: Kendall’s τ = 0.45, p < 0.001), indicating significant similarity between the pigment composition gradients captured by the two methods. Interestingly, chl *a* and its degradation product phe *a* were well separated by modified-GPS as indicated by the high correlation between methods for these pigments (r^2^ = 0.97 for both pigment pairs). Many carotenoids were, nevertheless, clearly misidentified compared to HPLC.

#### Natural lake samples

**Fig 5:**
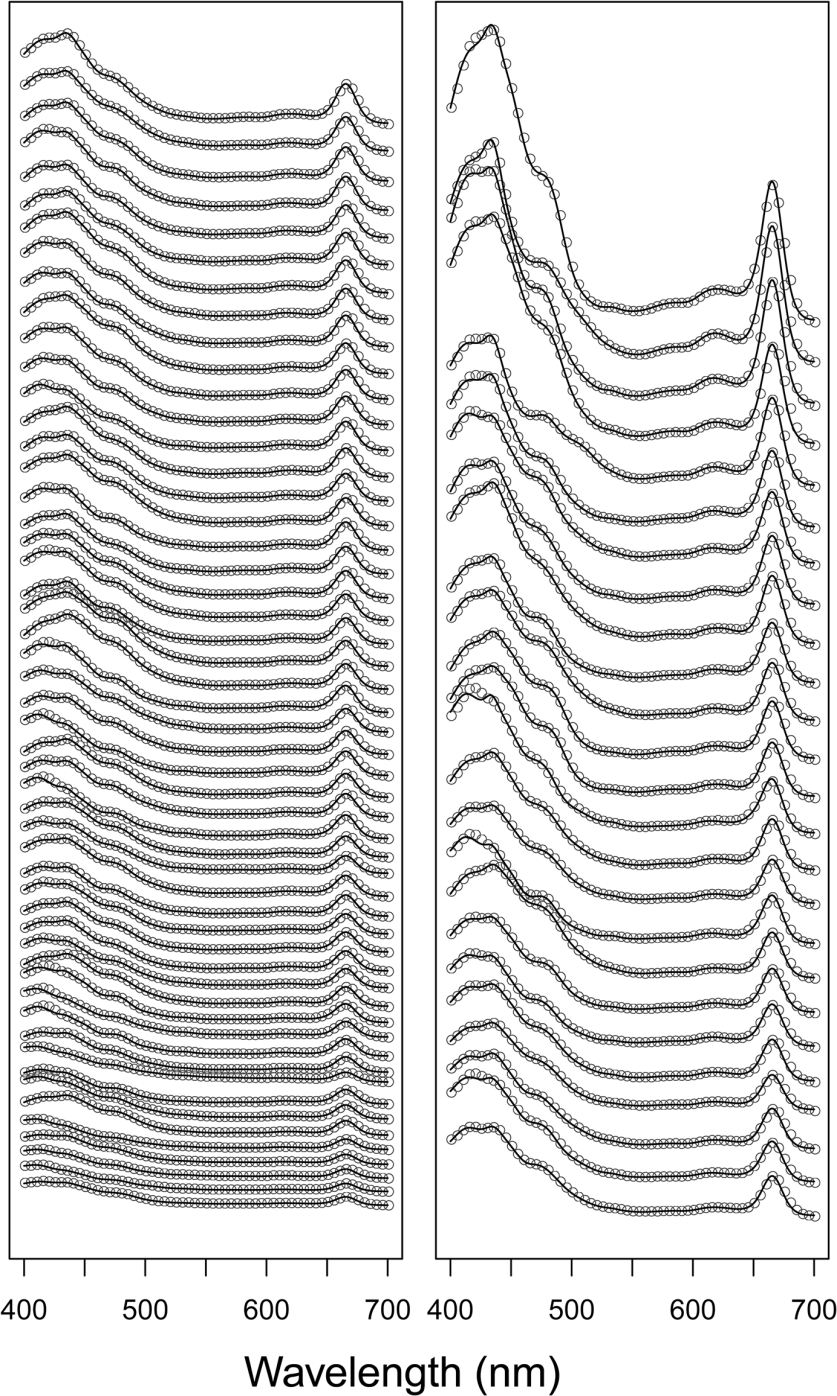
Observed and fitted spectra from lake samples. Absorbance spectra from the 75 natural lake samples (not normalized to unit path-length) plotted as grey dots. For clarity, spectra are ordered from lowest to highest maximum absorbance, and dots plotted only for every fifth nm. A vertical offset is added between each spectrum, therefore no units on the y-axis. Fitted spectra are added as black lines.

**Fig 6:**
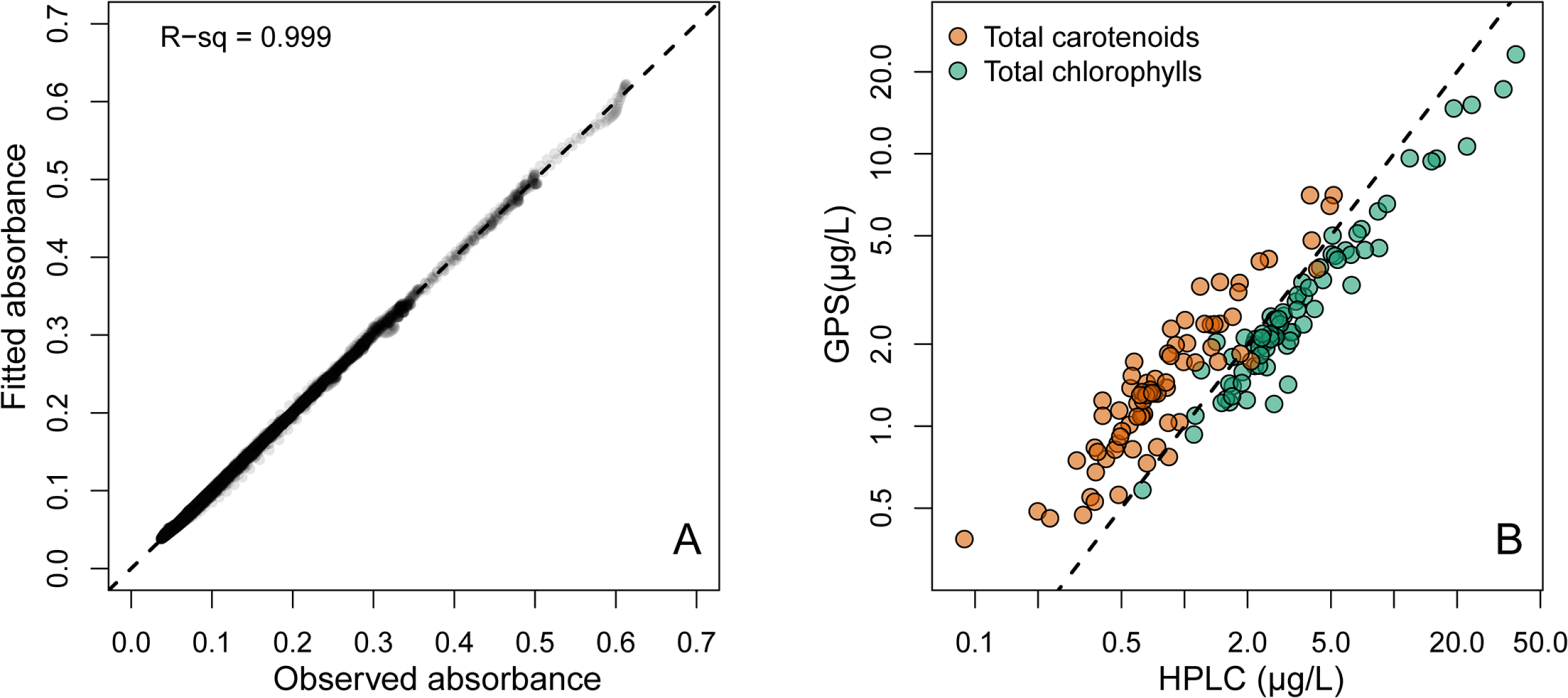
Total chlorophylls and total carotenoids in lake samples. A) Observed vs. fitted absorbance for all 75 spectra in Fig 5. r^2^ > 0.999. B) Concentrations of total chlorophylls (green) and total carotenoids (orange) estimated by modified-GPS (y-axis) and HPLC (x-axis). The dotted line shows the 1:1 relationship. Note that both axes are log-transformed due to the large variation in concentrations.

Spectra fitted by NNLS were again very similar to the observed natural lake sample pigment spectra (Fig 5 and Fig 6A), with RMSE values spanning from 0.0004 to 0.005 (mean = 0.0013). Estimates of total chl and total car by modified-GPS were highly correlated with HPLC (Fig 6B, log-log relationships: r^2^ = 0.94 for total chl, and 0.84 for total car, both p-values < 0.0001, n = 75). The modified-GPS method, however, underestimated total chl and overestimated total car (Fig 6B), compared to HPLC.

There was a significant correlation between scores on PCA axis 1 from the two methods (Kendall’s τ = -0.2, p < 0.05; notice that signs of PCA axes are arbitrary and without consequence), but not between PCA axis 2 scores. This indicates that the pigment compositions were similar for modified-GPS and HPLC, but that many individual pigment estimates, especially among carotenoids, were dissimilar.

#### Phytoplankton cultures

**Fig 7:**
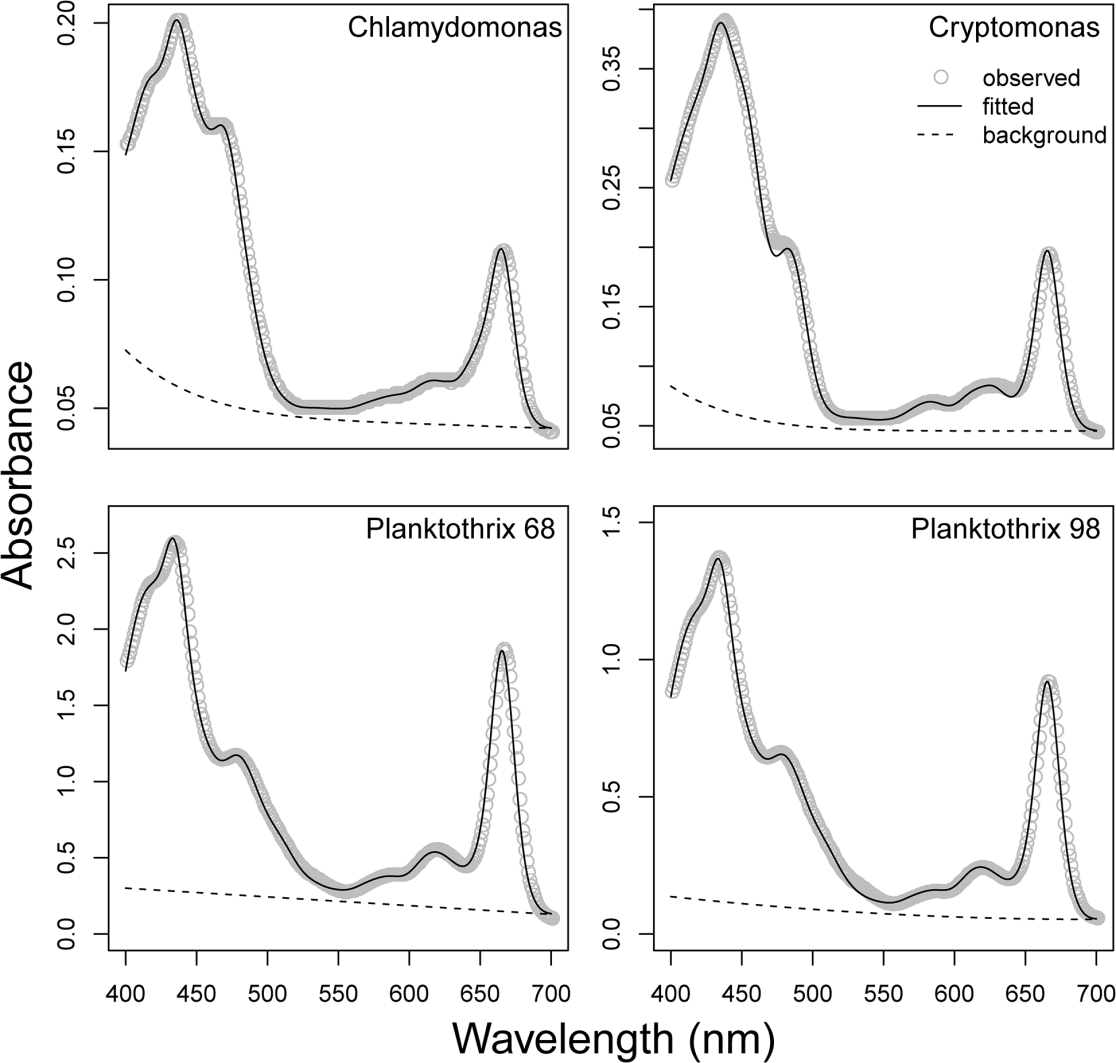
Observed *vs*. fitted spectra from culture samples. Observed absorbance spectra (grey dots) overlaid with fitted spectra (black lines) for four different phytoplankton cultures. Modelled background spectra are shown as dashed lines.

Observed and fitted spectra (Fig 7) were similar, with RMSE values ranging from 0.0008 for *Chlamydomonas* to 0.019 (Fig 7A) for *Planktothrix 68* (Fig 7C). Before comparing individual pigment estimates, we normalized all concentrations to chl *a* to avoid differences in extraction efficiency between methods. Due to the similarity between the zea and ββ-Car spectra, these two pigments were merged and presented as ββ-Car.Zea.

**Fig 8:**
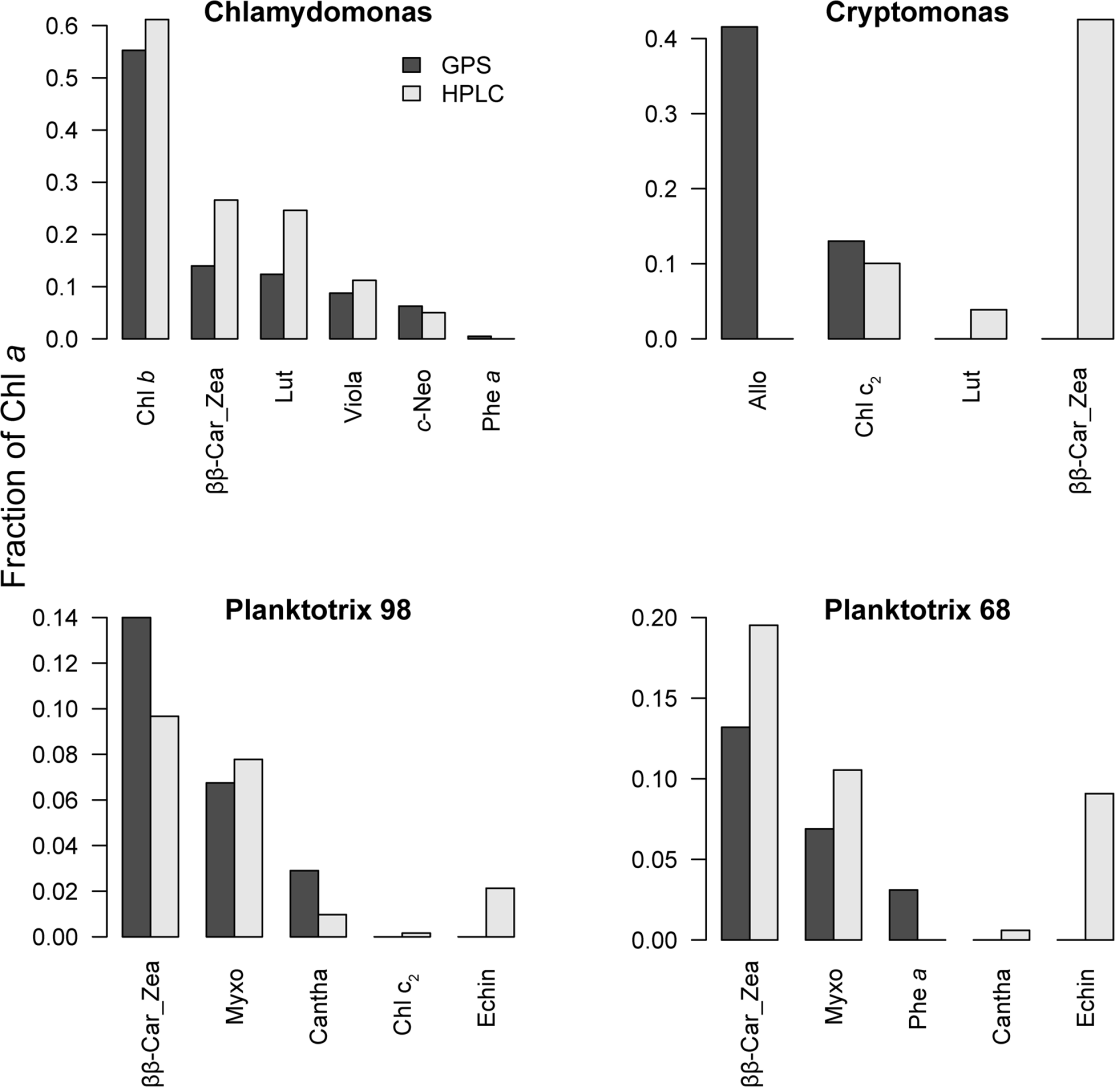
Pigment concentrations in culture samples. Concentrations of pigments in four phytoplankton culture samples presented as fractions of chl *a*. A) *Chlamydomonas reinhardii*, B) *Cryptomonas ozolinii*, C) *Planktothrix* 98, D) *Planktothrix* 68. Dark grey bars: modified-GPS, light grey bars: HPLC.

Concentrations of chl *b* and *c*_*2*_ were in good agreement with HPLC in *Chlamydomonas* and *Cryptomonas* (Fig 8A and 8B). Surprisingly, a small amount of chl *c*_*2*_ was identified by HPLC in the *Planktothrix* 98 strain (Fig 8C), even though this pigment should not be present in cyanobacteria. A small amount of phe *a* was identified by the modified-GPS in *Chlamydomonas* and *Planktothrix* 98, but standards for this pigment were not included in the HPLC analysis. For carotenoids, we observed larger differences between methods. In the *Chlamydomonas* sample concentrations were reasonably similar (Fig 8A). In *Cryptomonas*, modified-GPS identified allo, which is a cryptomonad-specific pigment, while HPLC identified ββ-Car.Zea as the dominant carotenoid (Fig 8B) based on the sample absorbance peaks at 427, 454, and 483 nm (482 in one replicate sample). These peaks were closest to the zea pigment standard peaks (428, 454, 480.9), but also close to the allo peaks (431, 455, 485). A small amount of lut was also identified by HPLC. In both strains of *Planktothrix* cultures, there was an inconsistency between methods for the carotenoid echinenone that was detected by HPLC, but not apparent in the modified-GPS. The comparison of *Chlamydomonas* pigment concentrations for samples extracted with either acetone and the Küpper et al. [14] method or ethanol with our modified-GPS were very consistent (data not shown).

#### Direct comparison of GPS and modified-GPS

Testing of the two methods using a single culture of the green algae *Chlamydomonas* with different extraction solutions resulted in no statistical differences (data not shown). When the Küpper et al. [14] method was used on the acetone extracts and our version on the ethanol extracts, the results from this test were very similar, indicating that each method works well on the correct solvent.

#### The background attenuation signal

**Fig 9:**
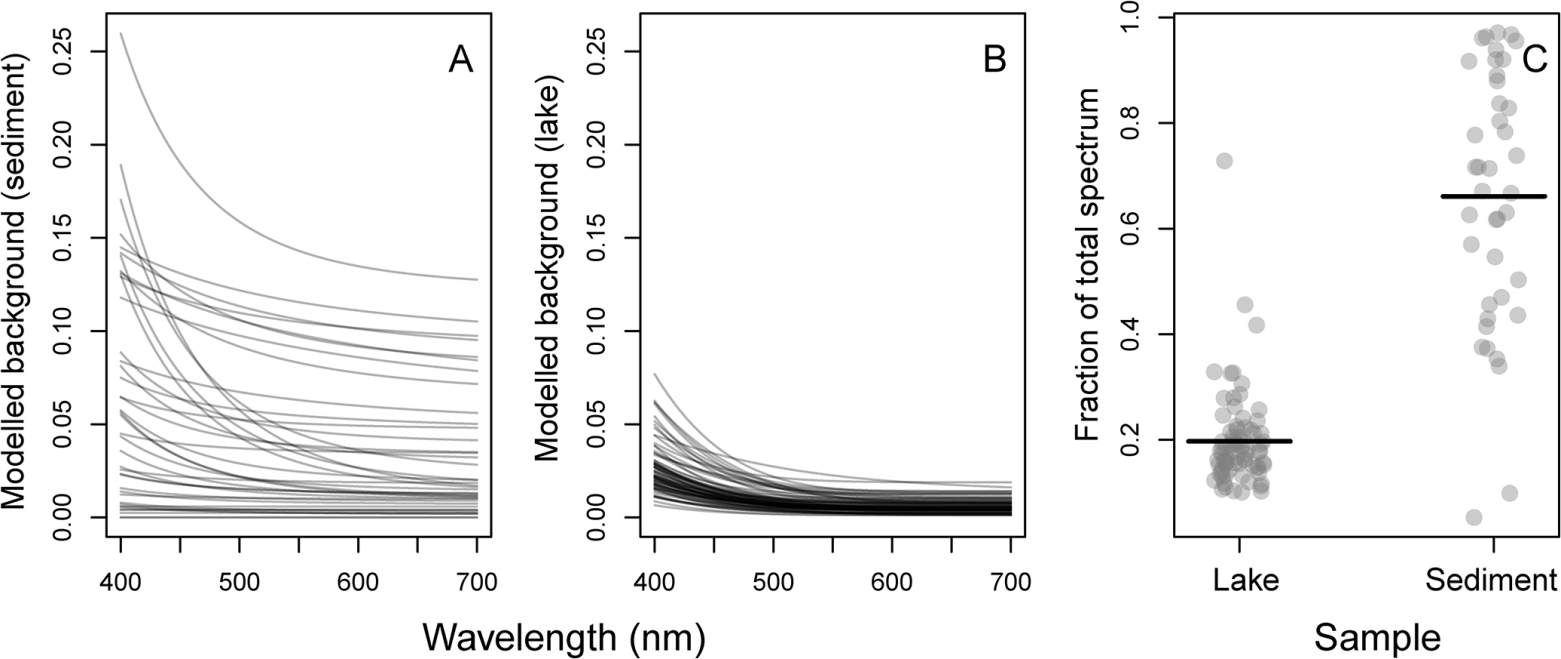
Modelled background spectra (scattering, and/or absorption by non-algal components) estimated after subtracting the microplate blank spectrum from the raw absorbance spectra. A) Sediment samples (n = 40) and B) natural lake samples (n = 75). Each grey line represents the modelled background spectrum from one sample. C) The fraction of the total spectrum constituted by the background spectrum. It was calculated by dividing the integral under each background spectrum by the integral under the corresponding total (pigment + background) spectrum. Horizontal line equals mean fractions. The background constituted a higher and more variable fraction in the sediment samples.

The modified-GPS method estimated a significant background signal on all the blank-corrected spectra for both sediment (Fig 9A) and lake (Fig 9B) samples. The fraction of the total spectrum constituted by the background spectrum, calculated by dividing the integral under each background spectrum (bg_int_) by the integral under the corresponding total (pigment + background) spectrum, was generally higher and more variable in the sediment samples compared to the lake samples (Fig 9C). A linear regression with log(bg_int_) as a function of log(POC) and log(chl a) revealed a significant positive relationship with chl *a* (p < 0.0001), but a non-significant effect of POC using natural the lake data (n = 75, r^2^ = 0.48). This indicates a contribution to the background from the amount of algae on the filter, possibly scattering by algae-related particles. No effect of POC might indicate that absorption by non-algal compounds matter is less important.

## Discussion

### Comparing the GPS with other spectrophotometric methods

Classical multi-wavelength spectrophotometric methods (e.g., [7], [8], [9]) all depend on subtracting a solvent blank or the absorbance at 750 nm, where all pigment spectra have low absorbance. Our results (Fig 9) show that the non-pigment background spectra are non-constant, and that their contribution to total absorbance can be substantial in natural samples, especially from sediments. Since the background spectra generally increase toward lower wavelengths, it means that especially carotenoids will be overestimated without proper background correction, while chlorophylls estimated from single-wavelength measurements will be less biased. It also means that carotenoid to chlorophyll ratios will be better estimated by modeling the full spectrum by actual pigment basis functions, than by empirical equations based on absorbances at a few wavelengths. Given the processing speed of modern scanning spectrophotometers using the microplate format, there are hardly any advantages of using the classical multi-wavelength methods any more.

### Comparing the GPS method with the modified-GPS method

The original GPS method [14] is conceptually sound but due to dependence on SigmaPlot, it is also functionally limited. Use of single-cuvette spectrophotometry and individual sample handling in SigmaPlot along with the need for separate compilation of data and pigment concentration determination makes the method cumbersome and time-consuming. By implementing into the open source, free software R, we make the method accessible to everyone and provide an environment where compiling of results, graphing, and additional analysis can rapidly and easily be executed by a few lines of code. As an example, use of plate technology allows 96 samples to be scanned, analyzed, and compiled into usable data in the time one sample can be processed by the original method. This results in a significant increase in analytical speed that allows for an expansion of the usefulness of the method.

Several conceptual modifications were implemented to better refine the computations. One was to change to NNLS. The advantage of using NNLS over non-linear optimization is primarily the efficiency of computation. Another critical modification was to replace the exponential model of the background spectrum by a weighted sum of basis functions. By doing so, both the pigment and background spectrum can be modelled efficiently and linearly in one step. These modifications allow for rapid fitting of pigment and background spectra, and calculation of pigment concentrations in a large number of samples simultaneously.

Although both GPS methods are able to determine pigment concentrations accurately, a direct comparison between the two is complicated because the modified-GPS is adapted to ethanol extracts and based on ethanol-derived standards, while the original uses acetone. Further, the original method misses several pigments that are common in natural lake and sediment samples (peridinin, dinoxanthin, alloxanthin, and pheophorbide *a*), and includes many pigments that are less likely to be found (e.g. zinc and cadmium substituted chlorophylls, aurochrome, several different *cis*-forms of zeaxanthin). The component pigment spectra would therefore differ between methods, and the choice of which pigments to include would be arbitrary. However, we tested the results of the two methods by extracting pigments from the same cultured green algae sample using either acetone or ethanol on separate replicate samples and then applied the two versions of the methods such that standard solvents would also match extraction solvents. The results from this test were very similar, indicating that each method works well on the correct solvent.

### Pigment selection

As mentioned, in the original paper, Küpper et al. [14] included GP spectra for many rare or unusual pigments, including Cd and Zn-substituted chls, and *cis*-forms of many carotenoids. While this can be useful in specific experimental settings (see for example Küpper et al., 2002 [40]) or when analyzing cultures where these pigments are known to be present, it introduces unnecessary confusion when analyzing natural pigment samples with unknown community composition. The method of pigment selection utilized in the modified-GPS was based on the statistical analysis of possible error rates that occur when using spectral fitting. Our analysis addressed pigment selection quantitatively to propose a minimum set of potentially identifiable pigments that also have a high likelihood of appearing in natural samples. Using a Monte-Carlo simulation allowed us to determine error rates for the full list of individual pigments, thus enabling a quantitative basis for the selection of the final set of 19 core pigments, which can be used in observational studies and environmental surveys of unknown community structure. In an additional simulation, these 19 spectra were included as potential components in the modeling of a single pigment spectrum with added white noise. The result revealed that all 19 pigments should theoretically be correctly identified (S1 Fig). However, it is important to keep in mind the potential for aliasing between certain pigments, especially carotenoids when using any GPS method. The close similarity of many carotenoid absorbance spectra is a challenge to spectral deconvolution techniques in general. Many pigment pairs have correlation coefficients that are >0.98 (e.g. ββ-Car *vs* zea, diadino *vs*. lut, allo *vs*. zea) such that different combinations of weights and pigments can yield almost indistinguishable total spectra. This would be the case regardless of whether the original GPS or the modified-GPS method was implemented. This should still be taken into consideration when interpreting the estimated concentrations regardless of method used.

### HPLC vs the modified-GPS method

Our results indicate that the fit between HPLC and modified-GPS pigment analyses was not as accurate for individual pigments as it was when pigments were pooled into total carotenoids and chlorophylls. Judging from the Monte-Carlo simulation, the modified-GPS (and probably the original GPS method) algorithm had particular problems separating different carotenoids, leading to high false negative and false positive rates when unknown samples were modeled using a set of candidate pigments (Fig 2). However, HPLC is also not without error. For example, a small amount of chl *c* was detected by HPLC in the cyanobacterial cultures, while the cryptophyte specific pigment allo [17] was confused with zea in the analysis of *Cryptomonas*. This is understandable, since it was treated as an unknown sample in the HPLC analysis rather than a culture with *a priori* known pigment composition. The modified-GPS method, on the other hand, correctly identified allo as being present in the sample. Variation in HPLC derived concentrations often occurs between laboratories. For instance, in an inter-calibration study of replicate HPLC results compared between laboratories, Latasa et al., 1995 [41] found significant variation between laboratories, and, not surprisingly, with greater variation reported between the carotenoids than the chlorophylls. Use of external standards and standard reference materials was highly recommended to improve intra-laboratory variation. Such quality controls should be included in future comparisons between GPS and HPLC.

### Future perspectives

Because of the rapid throughput capability of the modified-GPS method, it is particularly useful for designed experiments where a large number of replicates are needed to ensure sufficient statistical power. This includes experiments with natural populations, as well as pure cultures or single specimens. Nutrient limitation bioassays with natural phytoplankton populations are typical examples of the former [e.g., 42, 43, 44]. In these experiments treatment units are spiked with nutrients in a factorial design, and the growth response typically recorded as change in chl *a*. With very little extra effort, the modified-GPS method could give important extra information in such experiments. The problem of carotenoid aliasing means that the modified-GPS can probably not be used for full-scale pigment-based chemotaxonomy, such as CHEMTAX [3]. But the resolution for the major chlorophylls (*a*, *b*, *c*_1_ and *c*_2_) should be sufficient to resolve responses of cyanobacteria (only chl *a*), chlorophytes and euglenoids (chl *a* and *b*), and chromophytes (Heterokonts, Haptophytes, and Cryptophytes: chl *a* and *c*_1_/*c*_2_). As such, the modified-GPS method should give new opportunities for interpreting experiments indicating N and P co-limitation. As pointed out by Arrigo [45], such responses could result from perfectly balanced N and P limitation in all populations (“Biochemical co-limitation”), or that some populations are limited by N others by P (“Community co-limitation”). The modified-GPS method should be capable of discriminating the former from the latter, given that there are sufficiently contrasting chlorophyll signatures between at least some of the populations involved.

The carotenoid to chlorophyll ratio is a common response parameter in physiological experiments involving light acclimation (e.g. [46, 47, 48]) or oxidative stress (e.g. [49, 50, 51]). Many of these studies use HPLC-based pigment analysis, but some (e.g. [46, 49, 51]) use just a single- or three-wavelength spectrophotometric method, such as [11]. The modified-GPS method offers significant improvement over classical “trichromatic” spectrophotometric methods by explicitly modeling the background absorbance instead of assuming it to be equal to a solvent blank. We think that estimates of total chlorophylls and carotenoids, relevant for many types of physiological and toxicological studies, can be obtained at sufficient resolution, and at substantially lower cost, with the modified-GPS method. Subject to the limitations of strongly aliased carotenoids, we also think that many pigment-level responses can be resolved by GPS using appropriate pigment basis functions for the test organism in a designed experiment.

We hope that scientists will use, modify, and improve our software in a variety of new experiments with phytoplankton, macroalgae, and higher plants.

## Supporting Information

S1 Fig Monte-Carlo simulation of core pigment detectability. Figure showing the results from a simulation of the detectability of the core pigments. Each of the 19 spectra were added a small amount of white noise (mean = 0, standard deviation = 0.04), and modelled as a function of all the 19 component spectra using NNLS.
(PDF)

S2 Fig The effect of different well plate types on absorbance and pigment concentrations. We extracted pigments from a *Planktothrix* strain in 96% ethanol, and measured the absorbance in 320 μL of the same extract in three different 96-well-plates: one polystyrene plate with clear walls, one polystyrene plate with white walls, and one polypropylene plate with clear walls. A) Raw absorbance spectra (cm^-1^) were similar for both polystyrene plates, but generally higher in the polypropylene plate B) Blank spectra (absorbance of empty wells filled with 320 μL 95% etOH) revealed that the differences were due to higher absorbance by the polypropylene plate compared to the polystyrene plates (thin lines in B). Thick lines in B) represent the blank (or background) spectrum modelled by the modified-GPS method. The modelled background is higher than the measured, indicating additional loss of photons due to scattering or absorbance by non-algal material in the sample. C) Absorbance spectra of the pigment extract after subtraction of the modelled background spectrum. Note that the spectra are now essentially similar. D) Pigment concentrations in the extracts from the different plates as estimated by the GPS method. Concentrations are similar in all plate types.
(PDF)

S1 File Instrument calibration. An R-script for calibration of the spectrophotometer, with ancillary text files. Contains the following files: “Instrument calibration.R” (the actual R-script), “pigment.function.R” (a script containing various functions for running the modified-GPS method), “gaussian.peak.parameters.txt” (a text file with Gaussian peak parameter describing the pigment spectra), “chl.a.spectrum.etOH.txt” (a sample chl *a* absorbance spectrum measured in 96% ethanol), and “specific absorption coefficients.txt” (the weight-specific absorption coefficient for each pigment).
(ZIP)

S2 File Estimating Gaussian peak parameters from pigment absorbance spectra. An R-script with ancillary files. “gaussian.peak.fit.nnls.R” contains various functions for the actual fitting. “chl.b.fit.R” contains an example on how to fit a chl *b* spectrum, also contained in the folder (“chl.b.spectrum.etOH.txt”). A word document describes the statistical background (“GP estimation_stat_background”).
(ZIP)

S3 File Monte-Carlo simulation of pigment error-rates. Contains an R-script for running the simulation (“Simulation of summed spectra.R”), which loads a script containing the actual simulation function (“Spectrum.simulation.function.R”). Ancillary files includes a text file with GP parameters for all pigments included in the simulation.
(ZIP)

S4 File Modified-GPS analysis of sediment samples. The R-script “Sediments.R” contains code for fitting spectra and calculating pigment concentrations in sediment samples. Ancillary files include the measured absorbance spectra from sediments, sample ID’s, specific absorption coefficients, GP parameters, and the “pigment.function.R”, which contains all functions used in the modified-GPS analysis. The script “Sediments GPS vs. HPLC_PCA.R” contains the analysis of concentrations vs HPLC for sediment samples.
(ZIP)

S5 File Modified-GPS analysis of lake samples. The R-script “Lake.R” contains code for fitting spectra and calculating pigment concentrations in lake samples. Ancillary files include the measured absorbance spectra from lakes, sample ID’s, specific absorption coefficients, GP parameters, and the “pigment.function.R”. The script “Lake GPS vs. HPLC and PCA.R” contains the analysis of concentrations vs HPLC.
(ZIP)

S1 Text Description of functions. A document describing the various functions used for fitting data and calculating pigment concentrations (contained in “pigment.function.R”).
(PDF)
