# Supplementary figures and images for: Spectrophotometric Analysis of Pigments: A Critical Assessment of a High-Throughput Method for Analysis of Algal Pigment Mixtures by Spectral Deconvolution
Source: PLoS One. 2015 Sep 11;10(9):e0137645. doi: 10.1371/journal.pone.0137645 (PMC4567325; doi:10.1371/journal.pone.0137645)

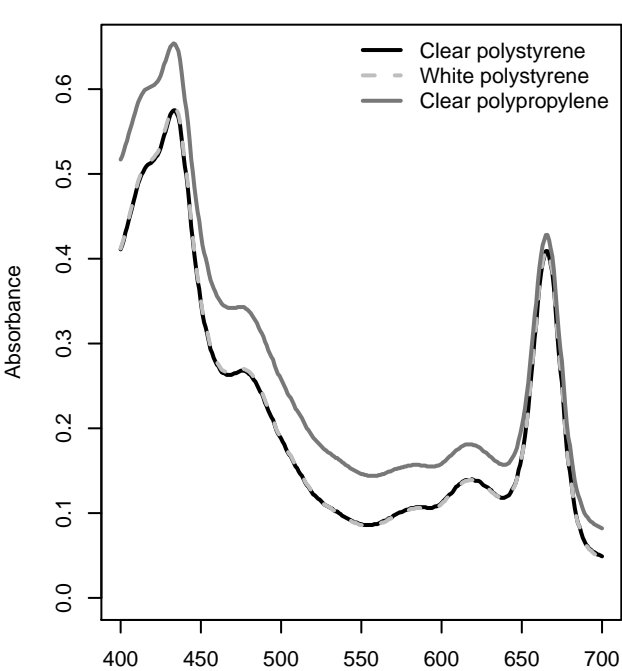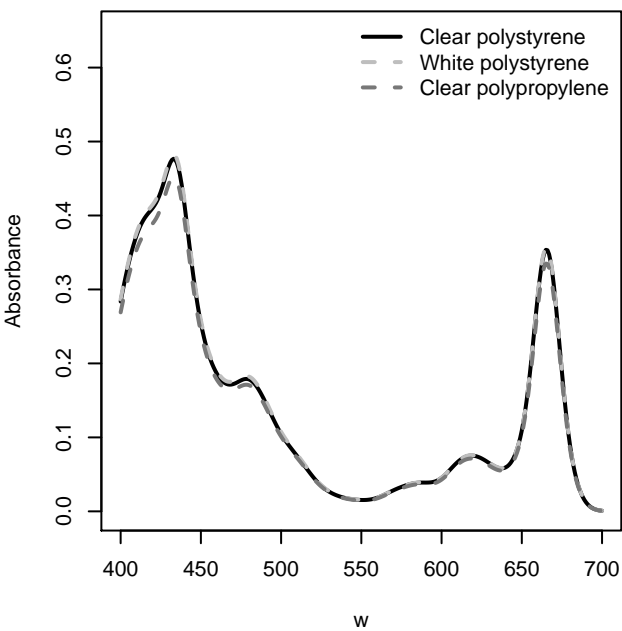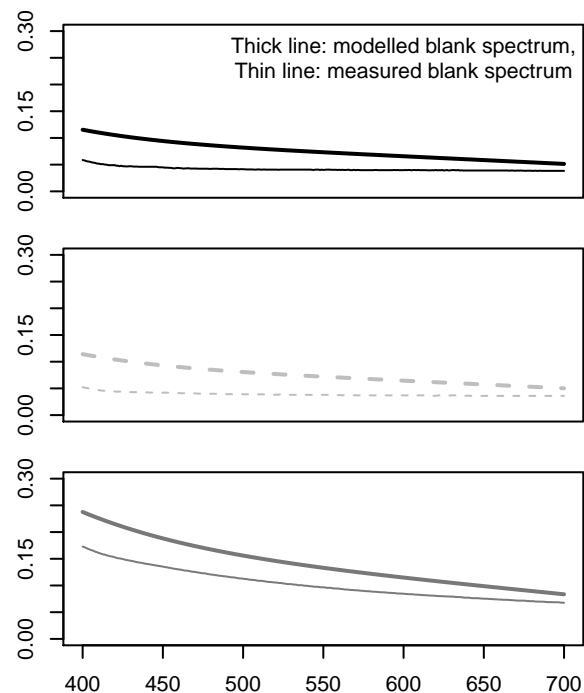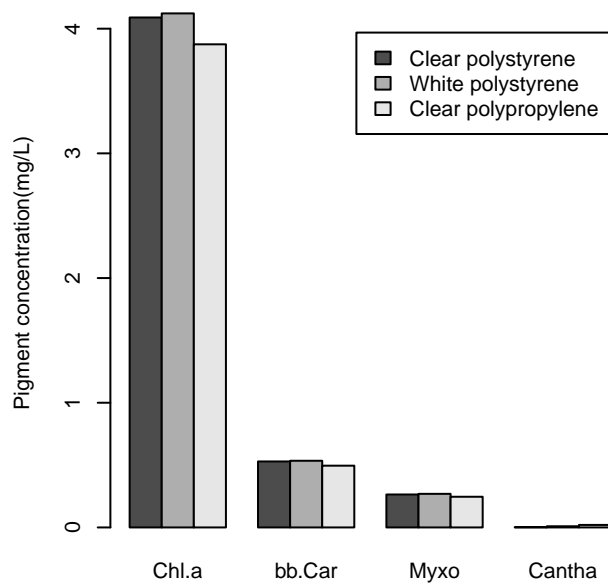

Supplement: S2 Fig — We extracted pigments from a Planktothrix strain in 96% ethanol, and measured the absorbance in 320 μL of the same extract in three different 96-well-plates: one polystyrene plate with clear walls, one polystyrene plate with white walls, and one polypropylene plate with clear walls. A) Raw absorbance spectra (cm-1) were similar for both polystyrene plates, but generally higher in the polypropylene plate B) Blank spectra (absorbance of empty wells filled with 320 μL 95% etOH) revealed that the differences were due to higher absorbance by the polypropylene plate compared to the polystyrene plates (thin lines in B). Thick lines in B) represent the blank (or background) spectrum modelled by the modified-GPS method. The modelled background is higher than the measured, indicating additional loss of photons due to scattering or absorbance by non-algal material in the sample. C) Absorbance spectra of the pigment extract after subtraction of the modelled background spectrum. Note that the spectra are now essentially similar. D) Pigment concentrations in the extracts from the different plates as estimated by the GPS method. Concentrations are similar in all plate types. (PDF) [file pone.0137645.s002.pdf]
