# Supplementary material for: Spectrophotometric Analysis of Pigments: A Critical Assessment of a High-Throughput Method for Analysis of Algal Pigment Mixtures by Spectral Deconvolution
Source: PLoS One. 2015 Sep 11;10(9):e0137645. doi: 10.1371/journal.pone.0137645 (PMC4567325; doi:10.1371/journal.pone.0137645)
Supplement: S2 File — An R-script with ancillary files. “gaussian.peak.fit.nnls.R” contains various functions for the actual fitting. “chl.b.fit.R” contains an example on how to fit a chl b spectrum, also contained in the folder (“chl.b.spectrum.etOH.txt”). A word document describes the statistical background (“GP estimation_stat_background”). (ZIP) [file pone.0137645.s004.zip › GP estimation_stat_background.docx]

**Estimating Gaussian peak parameters from pigment absorbance spectra**

Modeling a pigment spectrum as a sum of Gaussian peaks amounts finding parameter vectors *b* (weigths), *m* (peak positions) and *w* (half peakwidhs) (all of length *k*) such that the residual norm between the measured spectrum and the model prediction is minimized. In contrast to fitting to a sum of known component spectra, this problem becomes non-linear in the *m* and *w* parameters. Fortunately, we can take advantage of the problem being linear in the peak weight parameters (*b*) by using a so-called pseudo-linear fitting strategy. In this approach we fit only the 2 *k* non-linear parameters (*m* and *w*) by non-linear optimization, while the linear peak weight parameters are fitted by NNLS within each optimization step. In addition to reducing computational costs and increasing numerical stability, this strategy also has a desirable side effect of constraining the peak weights to non-negative values. The problem of finding the optimal number of peaks necessary to represent a spectrum thus becomes simplified in the sense that redundant peaks will have zero-valued weights. All non-linear optimization methods need starting values, which by the pseudo-linear approach is reduced to just specifying initial peak positions and widths. We chose to use initial peak positions evenly spaced over the wavelength range and identical initial half-peak widths equal to half the inter-peak distance.
