# Supplementary material for: Spectrophotometric Analysis of Pigments: A Critical Assessment of a High-Throughput Method for Analysis of Algal Pigment Mixtures by Spectral Deconvolution
Source: PLoS One. 2015 Sep 11;10(9):e0137645. doi: 10.1371/journal.pone.0137645 (PMC4567325; doi:10.1371/journal.pone.0137645)
Supplement: S1 Text — A document describing the various functions used for fitting data and calculating pigment concentrations (contained in “pigment.function.R”). (PDF) [file pone.0137645.s008.pdf]

Functions in the “pigment.function.R” script

1)

`pigment.basis(w, pigments=core, m.bias= 0, s.bias=1)`

This function generates the individual pigment basis spectra over a wavelength range, e.g. 400-700 nm, as a weighted sum of Gaussian peaks (GPs).

`w`: a vector of wavelengths

`pigments`: a character vector with the pigments for which to generate spectra. Default is the core pigments, that is c("Chl.a", "Chl.b", "Chl.c1", "Chl.c2", "Phe.a", "Phe.b", "Allo", "bb.Car", "Cantha", "Diadino", "Diato", "Dino", "Echin", "Fuco", "Lut", "Myxo", "c.Neo", "Peri", "Viola"). All pigments listed in the Gaussian peak text file can be included, or a subset of these.

`m.bias`: Instrument-specific parameter allowing for a constant offset in wavelength position. Can be obtained using the “instrument.calibration.R” script. Default value is 0.

`s.bias`: Instrument-specific parameter allowing for adjustment of Gaussian peak widths. Can be obtained using the “instrument.calibration.R” script. Default value is 1.

Example

```
source("pigment.function.R")

matplot(400:700, pigment.basis(w = 400:700, pigments =
c("Chl.a", "bb.Car", "Lut", "Echin"), m.bias = 0, s.bias = 1),
type = "l", ylab = "Abs")
```

---

2)

`background.basis(w,k=6)`

This function generates the background component spectra over a wavelength range *w*.

k: the number of background components to be generated. If k = 6, which is default, six power functions of u = (max(w) - w) / (max(w) - min(w)) is generated, with powers ranging from 1 to 6. A vector of ones (u^0) is always included, to be able to model a constant y offset.

Example

```
matplot(400:700, background.basis(w = 400:700, k = 6), type =
"l", ylab = "Abs")
```

---

3)

pigment.fit(w, y, pigments=core, k=6, m.bias= 0, s.bias= 1)

This function does the actual NNLS fitting of the measured absorbance spectra as weighted sums of pigment and background component spectra.

w: a vector of wavelengths

y: a data frame with the measured absorbance spectra as columns. If a single spectrum is to be analyzed, a vector with absorbance values can also be used.

pigments: a character vector specifying the pigments to include in the fit. The 19 core pigments (see above) are used as default, but any subset of available pigments can be used.

m.bias and **s.bias**: as above.

---

4)

a) fitted.spectrum(pigment.fit)

b) background.spectrum(pigment.fit)

c) pigment.spectrum(pigment.fit)

These three functions use the pigment.fit() object as input and calculates the a) fitted total spectrum, b) the fitted background spectrum, and c) the fitted pigment spectrum.

---

5)

`pigment.concentration(pigment.fit, pathl = 1)`

This function uses the pigment.fit() object as input, and calculates concentrations of the specified pigments in mg/L in the extract. This is done by dividing each pigment’s estimated weight by the weight-specific absorption coefficient and multiplying with 1000, after path-length normalization.

`pathl`: the pathlength in the microwell (or cuvette) in cm. Defauls value is 1.
