# Supplementary material for: Spectrophotometric Analysis of Pigments: A Critical Assessment of a High-Throughput Method for Analysis of Algal Pigment Mixtures by Spectral Deconvolution
Source: PLoS One. 2015 Sep 11;10(9):e0137645. doi: 10.1371/journal.pone.0137645 (PMC4567325; doi:10.1371/journal.pone.0137645)
Supplement: S1 Fig — Figure showing the results from a simulation of the detectability of the core pigments. Each of the 19 spectra were added a small amount of white noise (mean = 0, standard deviation = 0.04), and modelled as a function of all the 19 component spectra using NNLS. (PDF) [file pone.0137645.s001.pdf]

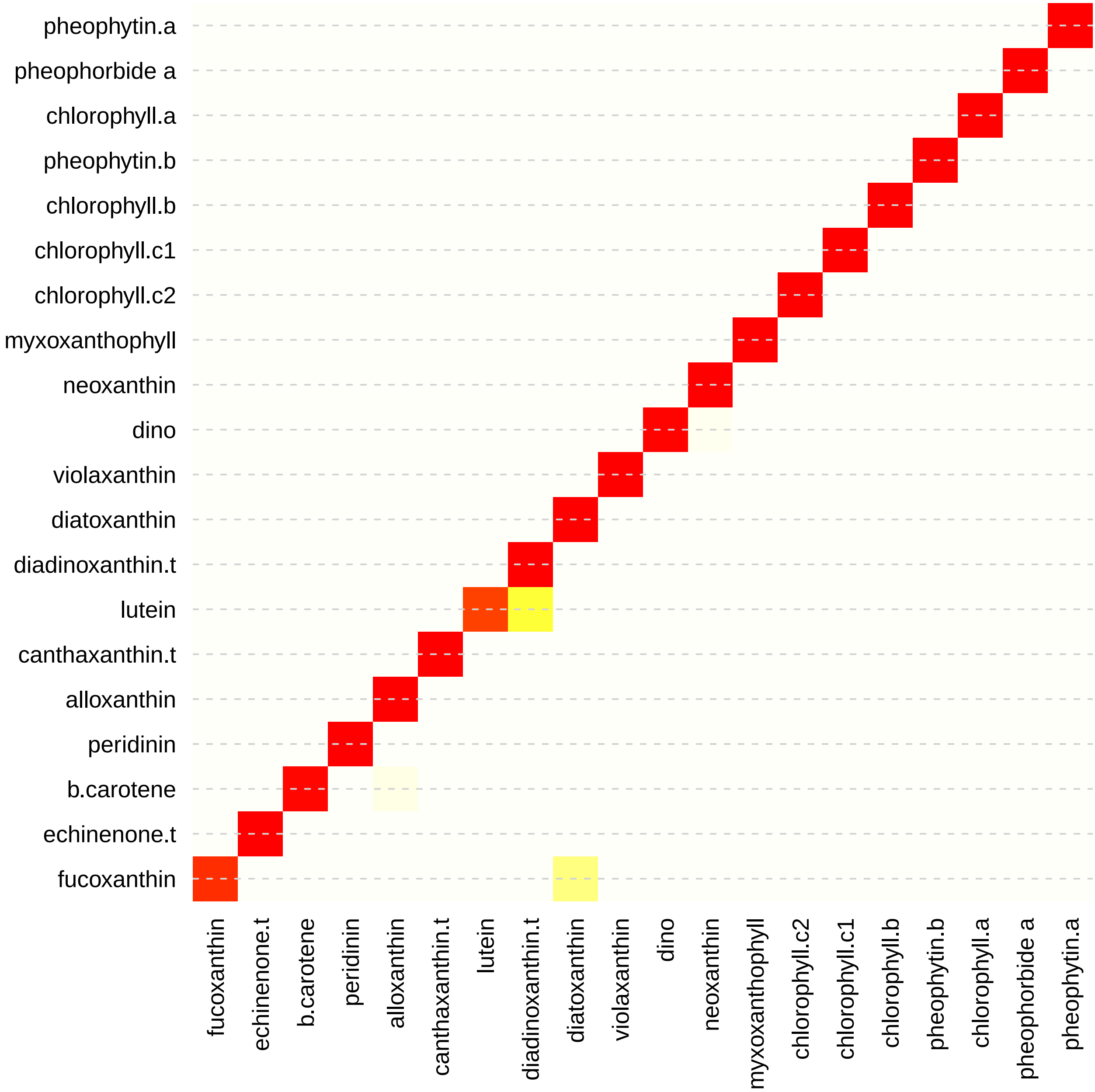
pheophytin.a
pheophorbide a
chlorophyll.a
pheophytin.b
chlorophyll.b
chlorophyll.c1
chlorophyll.c2
myxoxanthophyll
neoxanthin
dino
violaxanthin
diatoxanthin
diadinoxanthin.t
lutein
canthaxanthin.t
alloxanthin
peridinin
b.carotene
echinenone.t
fucoxanthin
fucoxanthin
echinenone.t
b.carotene
peridinin
alloxanthin
canthaxanthin.t
lutein
diadinoxanthin.t
diatoxanthin
violaxanthin
dino
neoxanthin
myxoxanthophyll
chlorophyll.c2
chlorophyll.c1
chlorophyll.b
pheophytin.b
chlorophyll.a
pheophorbide a
pheophytin.a
